# On the Use of a Depth Camera for the Assessment of Upper Extremity Movements in Healthy Individuals

**DOI:** 10.3390/s26061762

**Published:** 2026-03-11

**Authors:** Serkan Çizmecioğulları, Şenay Mihçin, Aydin Akan

**Affiliations:** 1 Biomedical Device Technology Program, Vocational School of Technical Sciences, Kırşehir Ahi Evran University, Kırşehir 40100, Türkiye; serkan.cizmeciogullari@ahievran.edu.tr; 2 Department of Mechanical Engineering, Izmir Institute of Technology, Izmir 35430, Türkiye; 3 Department of Electrical and Electronics Engineering, Izmir University of Economics, Izmir 35330, Türkiye; akan.aydin@ieu.edu.tr

**Keywords:** range of motion, stroke, rehabilitation, Kinect, reliability

## Abstract

Upper extremity impairments often lead to reduced joint range of motion (ROM), making reliable assessment essential for rehabilitation planning. This study investigated the within-day and between-day reliability of the Microsoft Kinect V2 depth camera for active upper extremity ROM assessment in 30 healthy adults. Ten predefined shoulder and elbow movements were recorded, and joint angles were computed using a custom vector-based algorithm. Within-day reliability ranged from moderate to excellent (ICC: 0.754–0.953), while between-day reliability ranged from moderate to good (ICC: 0.654–0.881). Absolute reliability varies substantially across movements. The SEM% values ranged from 2.1% to 17.3% within-day and from 2.8% to 23.6% between-day. The between-day MDC values were particularly high for certain movements (e.g., >20° for shoulder extension and >50° for elbow flexion), indicating limited sensitivity to detect small clinical changes. Additionally, shoulder adduction could not be reliably analyzed in 36.7% of participants due to self-occlusion-related tracking instability, highlighting a practical limitation of the Kinect V2 for certain upper extremity movements. These findings suggest that Kinect V2-based ROM assessment demonstrates acceptable reliability for large-amplitude planar movements under controlled conditions but shows substantial limitations for rotational and occlusion-prone tasks. The device may be suitable for research or screening applications; however, caution is warranted when interpreting small changes in clinical settings.

## 1. Introduction

The upper extremities of the human body consist of the shoulder, arm, and hands, providing functionality and motion in many daily life activities. Therefore, any disorder that causes upper extremity impairments has a negative impact on all aspects of one’s life [1]. There are a number of neurological disorders that cause upper extremity impairments. Spinal cord injury, multiple sclerosis, Parkinson’s disease, cerebral palsy, and stroke could cause motor impairments in the upper extremities. Among them, stroke is the second leading cause of disability worldwide [2]. About 80% of the stroke survivors experience upper extremity impairments [3]. Common upper extremity impairments after stroke are paresis, loss of fractionated movement, abnormal muscle tone, and changes in somatic sensations [4,5].

The problems mentioned above could cause reduced range of motion (ROM) and consequently result in a decrease in the life quality of an individual [6,7]. Therefore, proper treatment and rehabilitation are the main requirements for the recovery of stroke patients. In clinics, the measurement of ROM of a patient is very important to assess the initial level of joint restriction in the patient, decide on a proper treatment plan, evaluate the effectiveness of the treatment [8], and, finally, predict the recovery after stroke [9].

Currently, the ROM of joints is measured using goniometry or newer technological methods such as marker-based motion-capture systems (MBSs). Although goniometric measurement is cost-effective, its repeatability has been shown to be low [8]. On the other hand, MBS systems have high repeatability and accuracy, but they are expensive, requiring the use of multiple cameras and the time-consuming procedure of attaching markers to the body [10]. Because of these aforementioned problems, researchers are in search of repeatable, accurate, cost-effective, and easy-to-use alternatives.

In this context, it is also important to distinguish between active range of motion (AROM) and passive range of motion (PROM). AROM refers to voluntary joint movement produced by the individual’s own muscular effort, whereas PROM is achieved through external assistance and reflects joint mobility independent of neuromuscular control. Markerless depth-camera-based systems, including the Kinect V2, inherently capture only active, self-performed movements, as passive joint manipulation cannot be detected without external force application. Accordingly, the present study focuses exclusively on active range of motion, and all reported ROM measurements should be interpreted as functional, voluntary joint excursions rather than passive joint flexibility.

Among these alternatives, depth cameras may have the potential to provide a solution. As a type of depth camera, Kinect cameras have been considered as an option for the assessment of motion. There are some studies in which Kinect V2 has been used for the ROM assessment of lower extremities [11,12]. Similarly, it has been investigated whether the Kinect V1 camera could be used for upper extremity assessment. [13]. A study previously evaluated the reliability of Kinect V1 for shoulder abduction, elbow flexion, hip abduction, and knee flexion movements [14]. Quantified with the intraclass correlation coefficient (ICC) Kinect V1 provided ‘good’ relative reliability. Another study evaluated both the relative and the absolute reliability results of only four shoulder movements [15]. They concluded that Kinect V1 had ‘good’ (0.61-0.80) to ‘very good’ (0.61-0.80) ICC values for relative reliability and presented ‘good’ absolute reliability, except for the shoulder flexion at 90° ROM.

In another study that utilized a newer version of Kinect (Kinect V2), the within-day and between-day reliability analysis of Kinect V2 was investigated [16]. The within-day reliability of Kinect V2 was ‘good,’ but its between-day reliability was assessed as ‘poor’, since the participants could not repeat the movements with the same levels across days. In another study [17], conducted with Kinect V2, in the context of four functional tasks, the between-day reliability analysis of three shoulder movements and one elbow movement was examined. Kinect V2 presented high reliability. However, since these movements, which were assessed in this study, did not require the movement of joints with their full capacity, the reliability of Kinect V2 could not be assessed in full ROM.

Recently, ref. [18] analyzed the relative and the absolute reliability of five shoulder motions by considering the average of three days of measurements. They obtained ‘very good’ relative reliability results for shoulder flexion, shoulder internal-external rotation, shoulder abduction, and ‘good’ results for shoulder extension. In addition, Kinect V2 presented high absolute reliability. However, the study examined only the between-day reliability and did not consider the elbow movements. Previously, the agreement of the Kinect V2 with the gold-standard OptiTrack® (NaturalPoint Inc., Corvallis, OR, USA) motion-capture system was studied. In that study, Kinect V2 presented agreement with OptiTrack [19].

Although there is some research on the reliability of the Kinect V2 camera, there is a need for a comprehensive analysis of how reliable the Kinect V2 is for the purpose of ROM measurements in three anatomical planes. This study aims to assess the reliability of the Kinect V2 for upper extremity movements in these three anatomical planes as a potential tool for range of motion (ROM) assessment. Our ultimate goal is to develop a system that can be used to assess joint functionality in individuals who have had a stroke or are experiencing stroke-like symptoms. As a first step toward achieving this goal, we designed ten upper extremity movements to calculate the ROM in a healthy population using data collected by the Kinect V2. We intend to gather data to provide proof of concept, demonstrating that Kinect V2 data, when processed with a custom-made algorithm, can be reliably used calculate ROM for both within-day and between-day measurements. To this end, we designed an experiment to evaluate both the within-day and between-day relative and absolute reliability of the Kinect V2 across ten different upper extremity movements. These movements consisted of eight shoulder and two elbow joint movements.

## 2. Materials and Methods

### 2.1. Participants

In the in vivo study, in order to collect data from healthy volunteers, ethical committee permission was obtained, in line with the Helsinki Declaration from the Clinical Research Ethics Committee of Bezmialem Vakıf University (Ethical Application Ref: 54022451-050.05.04). The inclusion criteria for the participants were that they should not have had any previous or current musculoskeletal disorders. Twelve male and eighteen female volunteers participated in this study. The average age, weight, and height values for males were 30.4 ± 8,9 years, 80.3 ± 14.4 kg, and 177.4 ± 5.4 cm, and for females were 28.1 ± 9.6 years, 60.6 ± 7.3 kg, 163.3 ± 5.0 cm, respectively.

### 2.2. Data Acquisition

**Figure 1:**
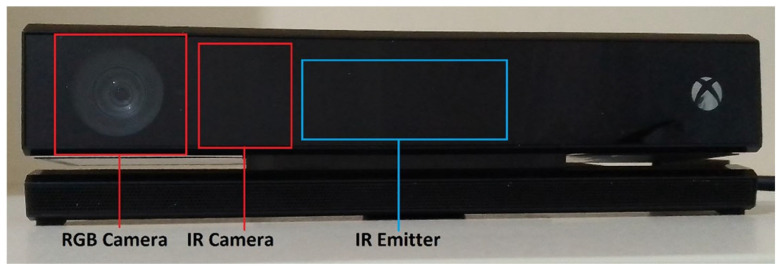
Kinect V2 camera components: RGB camera, IR camera, and IR emitter.

In this study, movement data were collected by the Kinect V2 (Microsoft Corp., Redmond, WA, USA) camera, which is a part of Xbox One Game Console. It consists of a Red, Green, Blue (RGB) color camera with a resolution of 1920 × 1080 pixels and 30 frames per second (fps) capturing rate, an infrared (IR) camera that has depth-sensing ability, with a data capturing rate of 30 fps, and an IR emitter (Figure 1). In this study, data from the infrared camera of Kinect V2 were used to calculate the ROM of the joints of interest. The Kinect depth camera has a 70.6° horizontal and 60° vertical field of view (FoV). The Kinect V2 utilizes a time-of-flight (ToF) technique and determines the depth by measuring the time in which the emitted light takes from the camera to the object and back [20,21]. Thanks to its depth-sensing ability with pattern recognition algorithms, the Kinect camera captures the 3D cartesian coordinates of 25 joints in the human body.

### 2.3. Experimental Procedure

**Figure 2:**
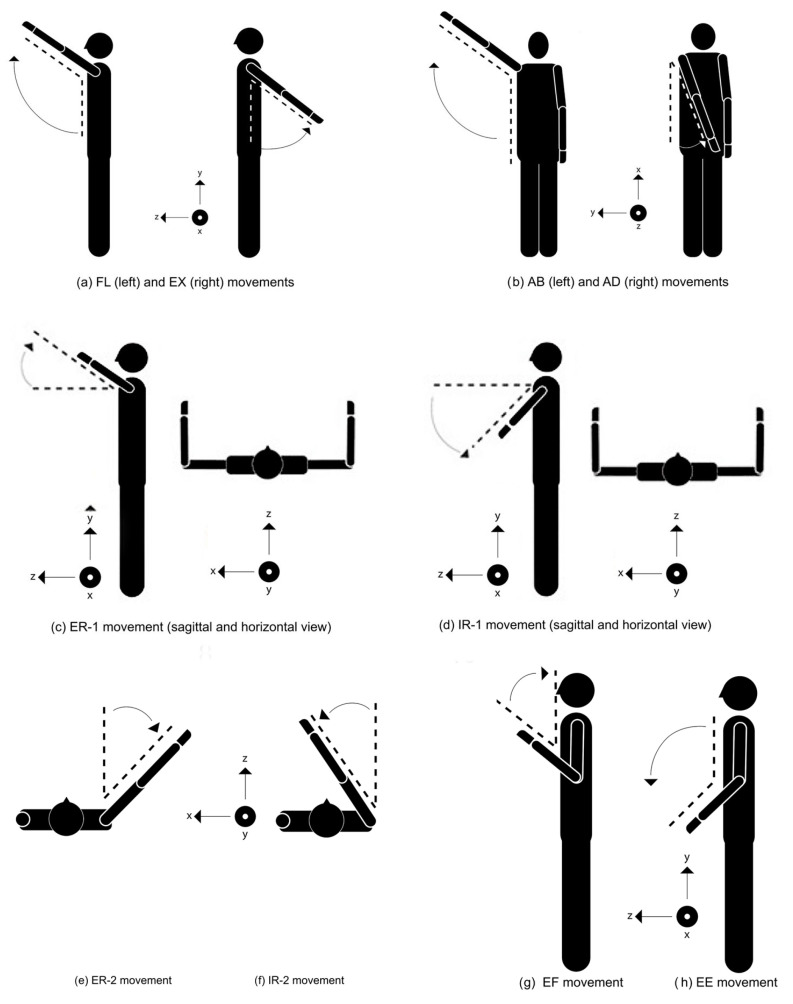
Shoulder and elbow movements in the sagittal, coronal, and transverse planes.

In this study, Kinect was placed facing the subject, at 2.0 m. The height of the camera was 0.75 m from the ground. The camera optical axis was aligned horizontally, with no intentional tilt, and the sensor was leveled prior to data collection. All movements were active, self-performed by the participants, and executed without external assistance. Before data acquisition, each movement was demonstrated once by the experimenter, and the participants were allowed one brief familiarization trial. Verbal cues were provided by the computer to initiate each recorded trial. In this study, individuals performed the requested movements twice within a week, with at least five days between each experimental day. In one day, each participant performed 10 types of movements (Table 1) which were repeated three times. These three repetitions were recorded as one trial. Using the 30 fps capturing rate of Kinect, 300 frames were recorded in each trial. The trials for the first day were named day 1 trials, and the trials for the second day were named day2 trials. In the following figures, for convenience in visualization, the subjects were depicted in standing position (Figure 2); however, during the study protocol, all movements were performed under following conditions: The subject sits on a seat without arm and back support. Knees are at 90° flexion and the hips are at 0° abduction while holding the trunk in an upright posture (i.e., there is no flexion and lateral bending of the trunk) constraining the trunk in a single plane. The arms are fully extended in parallel to the upper body. All the movements are performed using both right and left side limbs of the body simultaneously. However, only the dominant sides were considered in the calculations. The visual representations of all movements are shown in Figure 2. Each movement takes place in three anatomical planes: sagittal, transverse, and frontal.

### 2.4. Data Processing

All movements were recorded using the Kinect V2 camera. To collect and transfer motion data from the Kinect camera to the computer, a Graphical User Interface (GUI) was developed by writing custom-made code in Matlab^®^ (Mathworks Inc., Natick, MA, USA). The data collected with the depth camera was transferred to Matlab^®^ software. The 3D Cartesian coordinates (in units of meters) of 25 body joints were obtained using the Kinect depth camera. Initially, the data was filtered with a fourth-order Butterworth lowpass filter with a 1.5 Hz cutoff frequency. The selected cutoff frequency of 1.5 Hz was chosen based on the characteristics of the recorded movements and the study objective. The assessed tasks consisted of slow, controlled, self-paced active range-of-motion movements rather than rapid or ballistic functional actions.

The voluntary upper extremity ROM movements performed at self-selected speeds typically exhibit dominant frequency components below 2–3 Hz, whereas higher-frequency components largely reflect measurement noise and skeletal jitter inherent to depth-camera tracking. Accordingly, a low-pass filter was applied to attenuate noise while preserving the true movement signal. Similar low-pass filtering approaches within the 1–3 Hz range have been reported in kinematic analyses of slow upper extremity movements using markerless and motion-capture systems [22].

**Figure 3:**
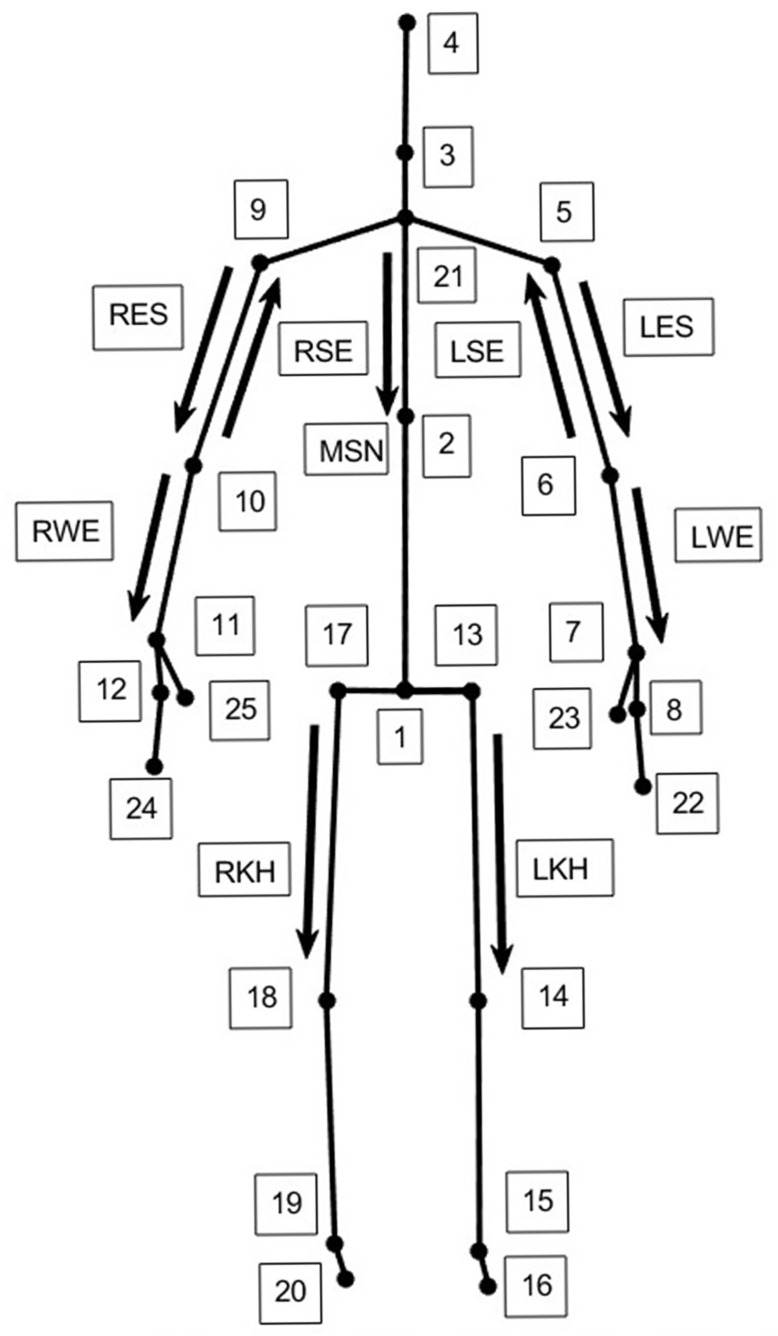
Kinect V2 skeletal view and the defined vectors shown in the frontal plane.

Therefore, the applied low-pass filter aimed to suppress sensor noise and skeletal jitter inherent to depth-camera tracking, while preserving the true movement signal. Similar cutoff frequencies have been reported in prior Kinect-based and markerless motion-capture studies focusing on joint angle estimation during slow upper limb movements. To eliminate the phase shift caused by filtering, the signal was filtered in both forward and backward directions. After the filtering stage, the movement vectors shown in Figure 3 were formed on the Kinect skeletal view. During the formation of vectors, 3D coordinates of related joints were used. Using these vectors the FL, EX, AB, AD, ER-1, ER-2, IR-1, IR-2, EF, and EE movement angles were calculated.

(Figure 3) illustrates the Kinect V2 skeletal model and the joint-based vectors used for joint angle computation. The depicted vectors represent the relative orientation between adjacent body segments and are constructed using the three-dimensional Cartesian coordinates of the corresponding joints. The visual elements shown in the figure do not represent procedural steps but rather illustrate the geometric relationships employed in the angle calculations. This schematic is provided to facilitate understanding of the vector-based approach used to estimate joint angles in different anatomical planes.

The joint angles for FL, EX, ER-1, IR-1, EF, and EE movements were calculated on the sagittal plane. For the AB and AD movements, the angles were calculated for the coronal plane. Finally, for ER-2 and IR-2, the movement angles were calculated for the horizontal plane. The angle *θ* between any pair of vectors *u* and *v* is calculated by the following relation:

(1) $$\theta =arctan\left(\frac{cross(u,v)}{dot(u,v)}\right)$$

where *cross(u,v)* is the vector product, and the *dot(u,v)* is the dot product of the two vectors. For the calculation of right and left FL and EX angles, the sagittal plane components of RES—MSN and LES-MSN vector pairs were used, respectively. For the right and left AB and AD angles, the coronal plane components of RES-MSN and LES-MSN vector pairs were used.

For the calculation of right and left ER-1 and IR-1 angles, the angle between the sagittal plane component of RWE and the projection of RKH vector on the sagittal axis (which is assumed to be parallel to the Kinect z-axis) of the body, and the sagittal plane component of the LWE vector and the projection of the LKH vector on the sagittal axis of the body were used. The reason for using projected vectors in these calculations is that the subjects were informed to hold their knees at 90° flexion and their hips at 0° abduction while holding the trunk in an upright posture; they showed some degree of deviation on the knee flexion and hip abduction angles. Therefore, the absolute peak values of the movements were changed. To eliminate this problem caused by the experimental procedure, the projected vectors were used in these calculations.

For right and left ER-2 and IR-2, the angle between the horizontal component of the RWE vector and the projection of the RKH vector on the sagittal axis (which is assumed to be parallel to the Kinect z-axis) of the body, as well as the horizontal component of the LWE vector and the projection of the LKH vector on the sagittal axis of the body were used.

For the calculation of right and left EF and EE angles, the sagittal plane components of RSE—RWE and LSE-LWE vector pairs were used, respectively. In the following section, we present a reliability analysis of both within-day and between-day ROM measurements by means of several statistical metrics.

### 2.5. Statistical Analysis

In this work, reliability of the measurements was assessed by the calculation of relative reliability and the absolute reliability indexes (Table 2). Relative reliability considers whether the subjects maintain their position in a sample over repeated measurements. This type of reliability is often assessed by the *ICC*. In this study, the *ICC (2,1*) model was used in the calculation of within-day reliability measurements and the *ICC (2,3*) was used in the between-day reliability measurements. The *ICC* ranges between 0 and 1. The level of relative reliability was quantified by values of the *ICC* as “excellent” (0.90-1.0), “Good” (0.75–0.90), “Moderate” (0.5–0.75), and “Poor” (0.0–0.5) [23].

On the other hand, absolute reliability examines the degree to which repeated measurements vary for subjects. If the variation between the subjects is low, the absolute reliability is high. The most commonly used absolute reliability indexes are: Standard Error of Measurement (*SEM*), *SEM%,* Minimal Detectable Change (*MDC*), and *MDC%.* These indexes are calculated with the equations below. The most common computation method for *SEM* is in the following form:

(2) $$SEM=SD\times \sqrt{1-ICC}$$

However, in Equation (2), *SEM* can be affected by the value of the *ICC*. Therefore, to avoid being dependent on the *ICC*, the following calculation method for *SEM* is preferred in this study [24]:

(3) $$SEM=\sqrt{{MS}_{E}}$$

where ${MS}_{E}$ is the Mean Square Error term from the repeated measures ANOVA analysis [25]. This section was removed in response to suggestions.

(4) $$SEM\%=\frac{SEM}{\bar{X}}\times 100$$

where $\bar{X}$ is the mean value of all measurements.

(5) $$MDC=Z_{score}\left(95\%\;CI\right)\times SEM\times \sqrt{2}$$

here the Z-score is 1.96 for a 95% confidence interval (CI), which is commonly used. The Z-score changes depending on the CI.

(6) $$MDC\%=\frac{MDC}{\bar{X}}\times 100$$

The values of *SEM* and *MDC* are expected to be low for reliable measurements. Lower *SEM* values indicate a lower measurement error and higher absolute reliability. *SEM%* expresses *SEM* relative to the mean, making it easier to compare across studies or variables. The *SEM%* values < 10% are considered “acceptable” [26]. The *MDC* is the minimal change that is attributable to the true change rather than to measurement error. The *MDC%* stands for the smallest change that indicates a real change in a single individual [22]. The parameter of *MDC%* provides information about the amount of relative random errors and sets a criterion for the acceptability of measurement agreement. In reliability studies, an *MDC%* < 30 is regarded as “acceptable”, and an *MDC%* ≤ 10 is regarded as “excellent” [27].

The interpretive thresholds used for *SEM%* and *MDC%* values (e.g., *SEM%* < 10%, *MDC%* < 30%) should be regarded as commonly used heuristic guidelines rather than universally accepted or normative criteria. These thresholds are context-dependent and may vary according to the studied population, movement type, and intended clinical or research application. In the present study, they are reported to facilitate comparison with prior reliability literature rather than to define absolute decision rules.

The reliability and agreement of the measurements in this study were evaluated as ‘within-day’ and ‘between-days’. For within-day calculations, day 1 trials were evaluated among themselves. The mean of day 1 and mean of day 2 trials were compared for between-day investigations. The statistical calculations were performed in SPSS^®^ 21 (IBM, New York, NY, USA).

## 3. Results

The results of our statistical analysis are presented in the Table 3 and Table 4. Although the total study population consisted of 30 participants, the shoulder adduction (AD) analyses were limited to 19 participants. In 11 participants (36.7%), the Kinect V2 failed to provide stable and physiologically plausible joint tracking during the AD trials. This tracking failure was primarily caused by self-occlusion and overlap of upper-limb segments, which resulted in inconsistent skeletal joint localization and unreliable ROM estimates across repeated trials. Consequently, these data were excluded from analysis.

The reliability analysis results for the dominant side of all participants on day 1 are presented in Table 3. In terms of relative reliability, Kinect V2 provides excellent results for the FL, AB, ER-1, and ER-2 movements and good reliability for the EX, AD, IR-1, IR-2, EF, and EE movements, as given in Table 3. Therefore, the Kinect V2 provides good to excellent relative reliability values for all movements. The highest *ICC* value is for the ER-2 movement, and the lowest *ICC* value is for the AD movement. In terms of absolute reliability, the Kinect V2 presents acceptable *SEM%* values for most of the movement types, excluding AD, IR-2, and EF. When it comes to *MDC%* values, the Kinect V2 presents excellent values for the FL, AB, and EE, acceptable values for the EX, ER-1, ER-2, IR-1, and unacceptable values for the AD, IR-2, and EF movements. The Kinect V2 presents the lowest *MDC%* value for the AB movement and the highest *MDC%* value for the AD movement.

Between-day reliability results are provided in Table 4 for the dominant side of the participants. In terms of relative reliability, the Kinect V2 presents good reliability for the FL, AB, ER-2, IR-1, EF, and EE movements and moderate reliability for the EX, AD, ER-1, and IR-2 movements. Therefore, the Kinect V2 provides moderate to good relative reliability values for all movements. The highest ICC value is for the FL movement, and the lowest *ICC* value is for the AD movement. When it comes to absolute reliability, the Kinect V2 produces acceptable *SEM%* values, excluding the EX, AB, ER-2, IR-2, and EF movements. The Kinect V2 produces excellent *MDC%* values for the FL and EE movements and acceptable values for the AB, ER-1, ER-2, and IR-1 movements, as shown in Table 4. In addition, the Kinect V2 produces the lowest *MDC%* value for the EE movement and the highest *MDC%* value for the AD movement.

As expected, both the absolute (*SEM*) and normalized (*SEM%)* error values increased from within-day to between-day measurements across all movements. This pattern reflects additional variability introduced by inter-session factors such as repositioning, movement replication differences, and day-to-day biological variation.

## 4. Discussion

In this study, the relative and absolute reliability of the Kinect V2 was assessed for both within-day and between-day measurements. Our results were compared with those of similar studies, as presented in Table 5. While our assessment included ten types of upper extremity movements, the studies listed in Table 3 considered fewer types of movements.

The observed reliability patterns are strongly movement-dependent and reflect both biomechanical and sensor-related factors. Planar shoulder movements such as flexion and abduction demonstrated higher reliability than rotational movements, which is consistent with prior depth-camera literature. Rotational tasks require inference of axial humeral rotation from relative joint positions, a process that is particularly sensitive to small joint localization errors. As a result, minor tracking inaccuracies may translate into disproportionately large angular deviations in rotational ROM estimates.

Self-occlusion represents a major source of measurement error in single-camera markerless motion-capture systems. During movements such as shoulder adduction and internal rotation, overlapping upper limb segments frequently obscure key joints from the camera’s viewpoint, leading to unstable skeletal reconstruction. The exclusion of the shoulder adduction data in a subset of participants reflects this known limitation and should be interpreted as a movement-specific tracking constraint rather than random data loss.

One of the key findings of this study is that Kinect V2 provides higher relative reliability for within-day measurements compared to between-day measurements. Our results support the findings of Study [16], which reported very good reliability for within-day measurements. However, in contrast to their results, our study demonstrates moderate to good reliability for between-day measurements.

Regarding relative reliability in the context of within-day measurements, our *ICC* values are comparable to those reported in Study [15] for the FL and ER-2 movements. In terms of absolute reliability, our study yielded lower *SEM* values for FL and ER-2 movements compared to their findings. However, due to the lack of *SEM%* values in the other studies, a direct comparison using this metric is not possible.

For between-day relative reliability, our *ICC* values are higher than those reported in Study [18] for the FL and EX movements, but lower for the AB, ER-2, and IR-2 movements.

Another notable finding is that the Kinect V2 demonstrates better absolute reliability in between-day measurements compared to within-day measurements. Although the *SEM* values for within-day measurements are lower than those for between-day measurements, the *SEM%* values are higher in the within-day context. All *SEM%* values in between-day measurements fall within the acceptable range. Similarly, the *MDC%* values for between-day measurements are lower than those for within-day measurements, further supporting the conclusion that the Kinect V2 exhibits better absolute reliability across different days.

When considering absolute reliability in between-day measurements, our *SEM* values for five types of movements are higher than those reported in Study [18]. However, as *MDC%* values were not provided in the other studies, comparisons based on this metric are not possible.

In within-day measurements, the Kinect V2 showed poor *MDC%* values for the AD, IR-2, and EF movements. These findings can be attributed to joint occlusion issues. Specifically, during SAD and EF movements, one joint may overlap with another, leading to inaccuracies in angle measurements.

Studies on single-camera RGB-D markerless motion-capture systems, including Azure Kinect–based pipelines, indicate that reliability is generally higher for large-amplitude planar movements and lower for complex, rotational, and occlusion-prone tasks [22,27]. These studies report that despite improvements in sensor hardware and body-tracking algorithms, self-occlusion and axial rotation remain persistent challenges across sensor generations. The present findings align with this evidence: the planar shoulder movements demonstrated higher reliability, whereas the shoulder adduction and rotational movements exhibited reduced reliability and increased tracking failure. This consistency across sensor generations suggests that movement complexity and camera viewpoint remain critical determinants of reliability, independent of specific depth-camera models.

As mentioned in Section 2.3, Experimental Procedure, projection vectors were used when calculating the angles. The use of projection vectors minimized individual-specific errors due to knee flexion and hip abduction and ensured that the reliability coefficients were closer to their true values.

While the use of projected vectors reduced variability caused by unintended deviations in lower-limb posture, this post-processing step effectively constrains kinematic degrees of freedom. As a result, projection may increase repeatability compared with unconstrained three-dimensional joint-angle calculations derived directly from raw joint coordinates. Therefore, the reported reliability values apply specifically to the projected-angle computation method, and reliability may be lower when using a full 3D joint-angle estimation without projection.

Although the Kinect V2 has demonstrated high reliability for planar shoulder movements such as flexion and abduction, the reliability of internal and external rotation measurements is more variable across studies. This variability is mainly attributed to the biomechanical complexity of shoulder rotation and inherent limitations of depth-camera–based motion capture. Shoulder rotation involves axial humeral motion, which cannot be directly measured by the Kinect V2 and must be inferred from relative joint positions, making rotational angles highly sensitive to small joint localization errors. In addition, self-occlusion during rotational tasks and the inability of the Kinect skeletal model to account for scapulothoracic motion further compromise tracking accuracy [28,29]. Consequently, while Kinect V2 may provide acceptable reliability for shoulder rotation in healthy individuals, these measurements should be interpreted with greater caution than planar shoulder movements. When the limb moves toward or away from the camera without a substantial change in frontal silhouette, depth-based skeletal inference becomes less stable, leading to increased variability in rotational angle estimates.

An important finding of this study concerns the magnitude of the Minimal Detectable Change (*MDC*) values. For several movements, particularly elbow flexion and shoulder extension, the between-day *MDC* values exceeded 20°, and in some cases 50°. Such magnitudes are substantially larger than clinically meaningful improvements typically expected in rehabilitation contexts.

In the stroke rehabilitation literature, clinically meaningful improvements in active ROM are often considerably smaller than the *MDC* values observed in this study. Therefore, the Kinect V2, in its current configuration, may lack sufficient sensitivity to detect small to moderate therapeutic changes at the individual level for certain upper extremity movements.

These findings suggest that while the device demonstrates acceptable relative reliability for ranking individuals within a group, its utility for detecting subtle longitudinal changes in clinical populations may be limited. Consequently, Kinect V2 should not be considered a replacement for established clinical measurement tools when precise change detection is required.

All in all, the reliability of the Kinect V2 was evaluated in terms of both relative and absolute reliability for within-day and between-day measurements. The device demonstrated good to excellent relative reliability for within-day assessments and moderate-to-good reliability for between-day assessments. The lower reliability observed between days may be attributed to participants’ inability to replicate movements with the same performance over a one-week interval.

Although reliability indices remained within acceptable ranges for certain movements, absolute error values increased between sessions, indicating reduced precision in longitudinal measurements.

Taken together, the findings suggest that Kinect V2–based ROM assessment is most reliable for large-amplitude, planar upper extremity movements performed under controlled conditions. In contrast, rotational movements and occlusion-prone tasks require cautious interpretation due to increased sensitivity to tracking errors. These considerations are critical when translating depth-camera-based ROM assessment in populations ranging from healthy individuals to clinical populations such as stroke survivors.

### Limitations

This study was conducted exclusively in healthy young adults, and the reported reliability results should not be assumed to directly generalize to stroke survivors. Post-stroke individuals frequently exhibit compensatory trunk movements, altered scapulothoracic coordination, spasticity, reduced movement speed, and intermittent pauses, all of which may challenge single-camera depth-based skeletal tracking differently than movements performed by healthy individuals. Accordingly, the present findings should be interpreted as reliability estimates for healthy populations, and future studies must explicitly evaluate reliability and tracking robustness in stroke cohorts.

An important limitation relates to the magnitude of measurement error. The relatively large *MDC* values observed in several movements indicate that the system may not be suitable for detecting small clinical changes in individual patients. This limitation is inherent to single-camera markerless motion-capture systems and should be considered when interpreting the findings.

Another important limitation of this study relates to movement-specific tracking instability. Shoulder adduction could not be reliably analyzed in 36.7% of participants due to self-occlusion and overlap of upper limb segments, which compromised skeletal joint localization. This represents a hardware- and algorithm-related constraint inherent to single-camera markerless motion-capture systems. Importantly, this limitation reflects a practical weakness of the Kinect V2, as certain upper extremity movements may not be consistently trackable across individuals. Such instability should be considered when evaluating the feasibility of using Kinect-based systems in clinical or research applications.

Although this study provides detailed relative and absolute reliability metrics, concurrent validity was not evaluated within the same dataset. Reliability reflects measurement consistency but does not establish agreement with a criterion standard. Because goniometric or optical motion-capture measurements were not collected concurrently, the present results demonstrate repeatability but cannot confirm measurement accuracy for each movement. Future work should incorporate simultaneous reference measurements to establish criterion validity alongside reliability.

## 5. Conclusions

This study evaluated the within-day and between-day reliability of the Kinect V2-based active upper extremity ROM assessment in healthy individuals. The system demonstrated moderate to excellent relative reliability for most movements, particularly large-amplitude planar tasks. However, absolute reliability varies substantially across movements, and large *MDC* values limit sensitivity for detecting small clinical changes.

Shoulder adduction could not be reliably analyzed in a considerable proportion of participants due to self-occlusion-related tracking instability, underscoring a movement-specific limitation of single-camera depth-based motion-capture systems.

Overall, the findings indicate that Kinect V2 may be appropriate for research applications, large-amplitude movement screening, or group-level comparisons under controlled conditions. However, caution is warranted when interpreting individual-level longitudinal changes, particularly for rotational or occlusion-prone movements.

Future research should focus on improving tracking robustness, validating findings in clinical populations, and exploring multi-camera or sensor-fusion approaches to reduce measurement error and enhance sensitivity to clinically meaningful change.

### Future Works

Our experimental results provide quantifiable evidence supporting the use of Kinect V2 for collecting ROM data from healthy individuals. This dataset offers valuable insight into the variance associated with both within-day and between-day measurements. These findings also lay the groundwork for future analyses focused on angular velocity, linear velocity, acceleration, and jerk behavior, particularly in stroke survivors. The results serve as a baseline for using deep learning algorithms to analyze movement patterns in post-stroke individuals as part of the next phase of our research.

## Figures and Tables

**Table 1:** Upper extremity movements and acronyms.

Acronym	Movement Description	Anatomical Plane
FL	Shoulder Flexion	Sagittal
EX	Shoulder Extension	Sagittal
AB	Shoulder Abduction	Frontal
AD	Shoulder Adduction	Frontal
ER-1	Shoulder External Rotation (Sagittal)	Sagittal
IR-1	Shoulder Internal Rotation (Sagittal)	Sagittal
ER-2	Shoulder External Rotation (Horizontal)	Transverse
IR-2	Shoulder Internal Rotation (Horizontal)	Transverse
EF	Elbow Flexion	Sagittal
EE	Elbow Extension	Sagittal

**Table 2:** Acronyms used in this study.

Acronym	Movement Description
ROM	Range of Motion
ICC	Intraclass Correlation Coefficient
SEM	Standard Error of Measurement
MDC	Minimal Detectable Change
ToF	Time-of-Flight
GUI	Graphical User Interface

**Table 3:** Within-day (i.e., day 1) relative and absolute reliability results for the dominant side of all participants.

Movement Type	Mean ± SD	ICC	SEM	SEM%	MDC	MDC%
Shoulder Flexion (N = 30)	173.9 ± 15.4	0.942 * (0.896–0.970)	3.674	2.1	10.2	5.9 *
Shoulder Extension (N = 26)	41.5 ± 8.4	0.843 # (0.727–0.920)	3.444	8.3	9.5	23.0 #
Shoulder Abduction (N = 30)	170.9 ± 13.0	0.931 * (0.879–0.964)	3.542	2.1	9.8	5.7 *
Shoulder Adduction (N = 19)	43.9 ± 14.1	0.754 # (0.559–0.886)	7.595	17.3	21.1	48.0 &
Shoulder External Rotation-1 (N = 30)	95.2 ± 11.4	0.903 * (0.831–0.949)	3.696	3.9	10.2	10.8 #
Shoulder External Rotation-2 (N = 30)	74.5 ± 16.4	0.953 * (0.916–0.975)	3.616	4.9	10.0	13.5 #
Shoulder Internal Rotation-1 (N = 26)	79.6 ± 12.8	0.872 # (0.772–0.936)	4.628	5.8	12.8	16.1 #
Shoulder Internal Rotation-2 (N = 30)	52.6 ± 16.6	0.794 # (0.653–0.891)	7.946	15.1	22.0	41.9 &
Elbow Flexion (N = 25)	30.6 ± 12.1	0.886 # (0.794–0.944)	4.254	13.9	11.8	38.5 &
Elbow Extension (N = 29)	171.3 ± 8.8	0.771 # (0.627–0.875)	4.366	2.5	12.1	7.1 *

**Note**: The symbol definitions for the ICC are: “*”: excellent, “#”: good, “&”: moderate, “!”: poor. The symbol definitions for MDC% are: “*”: excellent, “#”: acceptable, “&”: not acceptable.

**Table 4:** Between-day reliability results for the dominant side of all participants.

Movement Type	Mean ± SD	ICC	SEM	SEM%	MDC	MDC%
Shoulder Flexion (N = 30)	173.3 ± 14.7	0.881 # (0.767–0.941)	5.083	2.9	14.1	8.13 *
Shoulder Extension (N = 26)	42.1 ± 7.5	0.738 & (0.418–0.882)	4.780	11.3	13.3	31.6 &
Shoulder Abduction (N = 30)	170.2 ± 12.6	0.773 # (0.525–0.892)	7.664	4.5	21.2	12.4 #
Shoulder Adduction (N = 19)	43.4 ± 14.2	0.654 & (0.078–0.868)	10.284	23.6	28.5	65.6 &
Shoulder External Rotation-1 (N = 30)	95.2 ± 10.2	0.683 & (0.343–0.848)	6.826	7.17	18.9	19.8 #
Shoulder External Rotation-2 (N = 30)	72.7 ± 14.7	0.846 # (0.675–0.927)	7.739	10.6	20.5	28.1 #
Shoulder Internal Rotation-1 (N = 26)	79.7 ± 11.4	0.801 # (0.553–0.911)	4.628	5.792	18.5	23.2 #
Shoulder Internal Rotation-2 (N = 27)	53.0 ± 16.0	0.748 & (0.441–0.885)	10.258	19.3	28.4	53.6 &
Elbow Flexion (N = 25)	29.9 ± 10.0	0.780 # (0.503–0.903)	6.044	20.2	16.8	56.1 &
Elbow Extension (N = 29)	171.5 ± 8.0	0.755 # (0.474–0.886)	4.901	2.8	13.6	7.9 *

Note: The symbol definitions for the ICC are: “#”: good, “&”: moderate. The symbol definitions for MDC% are: “*”: excellent, “#”: acceptable, “&”: not acceptable.

**Table 5:** Comparison of the relative and absolute reliability of within-day and between-day measurements with other studies.

Authors	Kinect Version	Subjects	Within-Day ICC	Between-Day ICC	Within-Day SEM (°)	Between-Day SEM (°)
Reither [16]	Kinect V1, V2	1	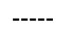	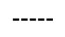	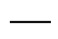	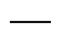
Bonnechere [14]	Kinect V1	48	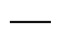	AB = 0.73 EF = 0.70	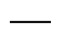	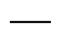
Huber [15]	Kinect V2	10	FL = 0.95 ER-2 = 0.98	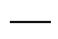	FL = 4.0 ER-2 = 3.7	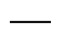
Çubukçu [18]	Kinect V2	40	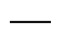	FL = 0.85 AB = 0.86 EX = 0.62 ER-2 = 0.87 IR-2 = 0.96	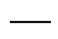	FL = 1.40 AB = 1.35 EX = 0.43 ER-2 = 1.98 IR-2 = 0.96
Our study	Kinect V2	30	FL = 0.94 ER-2 = 0.95	FL = 0.88 AB = 0.77 EX = 0.73 ER-2 = 0.84 IR-2 = 0.74 EF = 0.78	FL = 3.6 ER-2 = 3.6	FL = 5.083 AB = 7.664 EX = 4.780 ER-2 = 7.73 IR-2 = 10.25

“
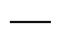
” indicates not reported in the source study. ICC models are reported as stated by the original authors. SEM values are in degrees.”
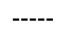
” indicates that a paired *t*-test was used in the study.

## Data Availability

The datasets presented in this article are not readily available because the data are part of an ongoing study.
